# Ameliorative Effects of Cardamonin on Monosodium Urate-Induced Gouty Arthritis through Inhibiting NLRP3 Inflammasome Mediation

**DOI:** 10.3390/medicina57090898

**Published:** 2021-08-28

**Authors:** Chih-Chien Wang, Jeng-Wei Lu, Yi-Jen Peng, Chian-Her Lee, Herng-Sheng Lee, You-Hsiang Chu, Chun-Jung Huang, Yi-Jung Ho, Feng-Cheng Liu, Chia-Chun Wu

**Affiliations:** 1 Department of Orthopedics, Tri-Service General Hospital, National Defense Medical Center, Taipei 114, Taiwan; tsghcc@gmail.com; 2 Antimicrobial Resistance Interdisciplinary Research Group, Singapore-MIT-Alliance for Research and Technology, Singapore 138602, Singapore; jengweilu@gmail.com; 3 Department of Pathology, Tri-Service General Hospital, National Defense Medical Center, Taipei 114, Taiwan; yijen0426@gmail.com (Y.-J.P.); olive_leaf@hotmail.com.tw (C.-J.H.); 4 Department of Orthopedics, School of Medicine, College of Medicine, Taipei Medical University Hospital, Taipei Medical University, Taipei 110, Taiwan; chianherlee@yahoo.com.tw; 5 Department of Pathology and Laboratory Medicine, Kaohsiung Veterans General Hospital, Kaohsiung 813, Taiwan; herngsheng131419@gmail.com; 6 Graduate Institute of Life Sciences, National Defense Medical Center, Taipei 114, Taiwan; joseph12825@gmail.com (Y.-H.C.); ejung330@gmail.com (Y.-J.H.); 7 School of Pharmacy, National Defense Medical Center, Taipei 114, Taiwan; 8 Rheumatologym, Immunology and Allergy, Department of Medicine, Tri-Service General Hospital, National Defense Medical Center, Taipei 114, Taiwan; lfc10399@yahoo.com.tw

**Keywords:** gouty arthritis, cardamonin, inflammation, chondrocyte, interleukin-1β

## Abstract

*Background and Objectives:* Gouty arthritis is an acute inflammatory response caused by the precipitation of monosodium urate (MSU) crystals in joints. The triggering of MSU leads to increased production of inflammatory cytokines, such as interleukin-1β, which in turn lead to the formation of macromolecular complexes, referred to as inflammasomes. Thorough characterization of the NLRP3 inflammasome can be used as an indicator of an immune response against harmful stimuli. Cardamonin is a chalcone, mainly found in the seeds of *Alpinia katsumadai*, and exhibits anti-inflammatory activity by inhibiting the release of pro-inflammatory cytokines in vitro. However, the mechanism by which cardamonin treatment alleviates gouty arthritis has yet to be fully elucidated. *Materials and Methods:* In vitro or in vivo models were used to study whether cardamonimn inhibited NLRP3 inflammasome activation or suppressed gouty inflammation. *Results:* In the current study, we determined that most NLRP3 was released passively after MSU stimulation, and this release of NLRP3 promoted caspase-1 activation and IL-1β secretion. Cardamonin was shown to decrease both the activity of caspase-1 and secretion of IL-1β in J774A.1 macrophage cells subjected to MSU stimulation. Cardamonin was also shown to attenuate the production of COX-2 in MSU-stimulated J774A.1 macrophage cells. Finally, cardamonin reduced the thickness of the synovial lining and the infiltration of gouty arthritis in a rat model. *Conclusions:* Overall, cardamonin significantly attenuated IL-1β secretion, caspase-1 activity, and COX-2 production stimulated by MSU. These findings provide new insights into the molecular mechanisms underlying the effects of cardamonin treatment for gouty arthritis.

## 1. Introduction

Cyclooxygenase-2 (COX-2) is a pivotal enzyme involved in maintaining inflammation through the catalysis of prostaglandin E2 and has been linked to the development of autoimmune disorders, coronary artery disease, and cancer [1,2]. COX-2 appears to be the dominant source of prostaglandins in inflammation and no doubt contributes to the development of acute inflammation in gouty arthritis attacks. At present, medications for the treatment of gouty inflammation include colchicine, nonsteroidal anti-inflammatory drugs (NSAIDs), steroids, and IL-1β blockers. However, the usage of NSAIDs raises safety concerns pertaining to patients with comorbid renal, cardiovascular, prostaglandin, or gastrointestinal conditions [3,4]. Thus, new pharmacological therapeutic strategies for the prevention of gout are required.

Gouty arthritis is an acute inflammatory response resulting from the precipitation of monosodium urate (MSU) crystals in joints. MSU can activate both innate and adaptive immune responses. COX-2, interleukin 8 (IL-8), and inducible nitric oxide synthase (iNOS) gene expression is triggered by MSU crystals in mononuclear cells and articular chondrocytes [5,6]. In an animal model of acute gout, the injection of MSU crystals was shown to trigger the production of proinflammatory cytokines, such as interleukin-1β (IL-1β) [7,8]. The release of IL-1β as a key regulatory proinflammatory cytokine in gout cases has been shown to promote the influx of neutrophils into the synovium and joint fluid, which is a pathological hallmark of an acute inflammatory attack [9]. MSU crystals upregulated genes’ expression of COX-2 and IL-8 in human synovial fibroblasts [10]. Moreover, IL-1β is produced in an inactive form by immune cells, which can be cleaved by caspase-1 to assume the active form [11]. Some evidence also shows that caspase-1 activates pro-IL-1β as well as pro-interleukin-18 (pro-IL-18) and converts them into active IL-1β and interleukin-18 (IL-18), respectively [12,13]. The activation of caspase-1 and IL-1β under MSU deposition causes the formation of macromolecular complexes, referred to as inflammasomes [14]. One of the most intensively studied inflammasomes is the nucleotide-binding oligomerization domain and leucine-rich repeat pyrin 3 domain (NLRP3) inflammasome, which play important roles in various inflammatory diseases. Once activated, NLRP3 upregulates cellular synthesis and the maturation of a variety of pro-inflammatory cytokines and chemokines such as IL-1β and IL-18, leading to inflammation against environmental or host-derived antigens [15]. In addition, the NLRP3 inflammasome can be used as an indicator of an immune response against harmful stimuli [16,17]. Blocking the expression of the NLRP3 inflammasome is an efficient approach in dealing with inflammatory diseases by a small-molecule inhibitor, MCC950 [18]. It has also been shown to mediate MSU-stimulated inflammation using a murine model of gout via NLRP3 inflammasome-mediated neutrophil recruitment and hypernociception depending on leukotriene B(4) [19]. On the other hand, the latest European Guidelines of Arterial Hypertension have introduced the assessment of uric acid into cardiovascular risk factors, which can be used as an independent predictor of all-cause and cardiovascular mortality, myocardial infarction, stroke, and heart failure [20].

Cardamonin (2′,4′-dihydroxy-6′-methoxychalcone) is a chalcone isolated from Zingiberaceae plants and reported to possess anti-inflammatory and antioxidative properties. Researchers have noted that cardamonin protects against lipopolysaccharide-induced acute lung injury and cisplatin-induced renal injury [21,22,23]. It has also been shown to inhibit colonic neoplasia, gastric cancer, and tumorigenesis by inhibiting the nuclear factor-kappa B (NF-κB) signaling pathway or modulating microRNA expression [24,25,26]. In recent studies, cardamonin has demonstrated inhibitory effects on the NLRP3 inflammasome in animal models of human diseases [27,28]. Our previous studies have shown cardamonin as a candidate nuclear factor E2-related factor 2 (Nrf2) activator for the treatment and prevention of osteoarthritis associated with inflammation and oxidative stress [29]. Nonetheless, the direct effects of cardamonin treatment on gouty arthritis have not been investigated.

The aims of this study was to assess the protective effects of cardamonin on chondrocytes and macrophages in gouty arthritis models. Our ultimate goal was to determine whether cardamonin could be used to ameliorate MSU crystal-mediated arthritis and to identify the mechanisms underlying the effects of cardamonin on synovial fibroblasts and articular chondrocytes in vitro and in vivo. In the future, it is also hoped to elucidate the signaling functions of cardamonin for the future development of targeted inflammation therapeutics.

## 2. Materials and Methods

### 2.1. Cell Culture

Murine monocyte-macrophage (J774A.1) cells were obtained from the American Type Culture Collection (ATCC, Manassas, VA, USA). J774A.1 macrophage cells were cultured in Dulbecco’s modified Eagle’s medium (DMEM) high-glucose medium (Gibco, Carlsbad, CA, USA) supplemented with 10% fetal bovine serum (FBS) (Gibco, Carlsbad, CA, USA), 2 mM L-glutamine, 10,000 U/mL penicillin, 10 mg/mL streptomycin, and 25 μg/mL amphotericin at 37 °C in a 5% CO_2_ incubator. Cells were subcultured twice weekly using standard protocols [30].

### 2.2. Cell Viability

The cell viability of murine J774A.1 macrophage cells was identified via an MTT assay. J774A.1 macrophage cells were seeded in a 96-well plate in 200 μL complete medium at a density of 10,000 cells per well. At confluence, J774A.1 macrophage cells were serum-starved overnight and then treated with various concentrations of cardamonin (0, 0.1, 1, 5, and 10 μM/mL) (Catalog number: C8249; Sigma-Aldrich, St. Louis, MO, USA) in serum-free medium for 6 h or 12 h. Briefly, J774A.1 macrophage cells were seeded in 96-well plates at a density of 1 × 10^4^ cells/well. Macrophage cells were further treated with 20 μL of 0.5 mg/mL MTT (Sigma-Aldrich, St. Louis, MO, USA) for 3 h, and formazan crystals (Sigma-Aldrich, St. Louis, MO, USA) were then solubilized with 150 μL of dimethyl sulfoxide (DMSO) (Sigma-Aldrich, St. Louis, MO, USA). Absorbance was recorded at 570 nm using the Synergy HT plate reader (Bio-Tek Instruments Inc., Winooski, VT, USA) [30].

### 2.3. Protein Extraction and Western Blot Analysis

Cells were immediately washed using ice-cold PBS and lysed in situ for 15 min with ice-cold RIPA lysis buffer (Thermo Fisher Pierce, Waltham, MA, USA) containing 100 μM Na_3_VO_4_ and 100X protease inhibitor cocktail (Thermo Fisher Pierce, Waltham, MA, USA). Cell lysates were collected after centrifugation at 13,000 rpm for 15 min, and the protein concentration was determined using the Lowry method. Cell culture medium was stored at −80 °C immediately after harvesting. Equal quantities of protein were then loaded onto 10% SDS-polyacrylamide gel and transferred to polyvinylidene fluoride (PVDF) membranes (Merck KGaA, Darmstadt, Germany). Membranes were blocked overnight at 4 °C with 2% BSA in TBST (12.5 mM Tris/HCl, pH 7.6, 137 mM NaCl, 0.1% Tween 20). After washing three times with TBST, blots were incubated with primary antibodies diluted in TBST. Specific antibodies were purchased from the following commercial suppliers: IL-1β, Catalog number: 4499 (Cell Signaling Technology, Danvers, MA, USA); NLRP-3, Catalog number: AG-20B-0014 (AdipoGen Life Sciences, San Diego, CA, USA); caspase-1, Catalog number: 4199 (Cell Signaling Technology, Danvers, MA, USA); COX-2, Catalog number: RB-9071 (Abcam, Cambridge, UK); and β-actin, Catalog number: sc-47778 (Santa Cruz Biotechnology, Dallas, TX, USA). After washing three times with TBST, the blots were incubated with HRP-labeled secondary antibodies for 1 h at room temperature. Membranes were rewashed thoroughly, after which binding was detected using the enhanced chemiluminescence plus western blotting detection system (Merck), in accordance with the manufacturer’s instructions. The membranes were scanned and analyzed via densitometry (VisionWorks LS, UVP, CA, USA), in accordance with the manufacturer’s instructions [29,30].

### 2.4. MSU-Induced Gouty Arthritis Model

Male Sprague Dawley (SD) rats were obtained from BioLASCO Taiwan Co., Ltd. (Yi-Lan, Taiwan). All experiments were approved by the National Defense Medical Center institutional animal care and use committee for the treatment of laboratory animals (Protocol No. IACUC-21-068). After seven days of acclimatization, 8-week-old rats were randomly divided into four groups: (1) control group; (2) MSU group; (3) prevention group; and (4) treatment group. Rats in the MSU group underwent intra-articular injection of MSU crystals into the knee joint (100 μL of 1 mg/mL MSU crystals) at 24 h prior to sacrifice. Rats in the prevention group underwent intraperitoneal injection of cardamonin (2.5 mg/kg) every day for five days followed by an intra-articular injection of MSU crystals into the knee joint at 24 h prior to sacrifice. Rats in the treatment group underwent an intra-articular injection of MSU (100 μL of 1 mg/mL MSU crystals) and then, 6 h later, underwent an intraperitoneal injection of 50 μL of 10 μM of cardamonin at 18 h prior to sacrifice [30,31].

### 2.5. Histology

The knee joints were fixed in 10% formalin, decalcified, trimmed, and embedded in paraffin blocks. Sections were prepared from tissue blocks and stained with hematoxylin and eosin (H&E) for histologic examination. H&E-stained slides were used for grading according to two synovial membrane features (thickening of synovial lining cell layer and inflammation) based on an assessment of synovitis scores, as previously described by Krenn [31,32]. Scores ranged from 0 to 6, with samples divided into those with low-grade (1–3) and high-grade (4–6) synovitis (Table 1). Note that synovitis scores could be used in the diagnosis of joint diseases.

### 2.6. Statistical Analysis

All values were expressed as mean ± standard deviation (SD) from at least three independent experiments. Statistical evaluation of the quantification data was performed using the ANOVA test and Dunnett post hoc test was chosen for comparing two different treatment groups, allowing for nonsymmetrical distribution data. The results were considered significant at a *p*-value < 0.05.

## 3. Results

### 3.1. The Cytotoxicity of Cardamonin on J774A.1 Cells

**Figure 1:**
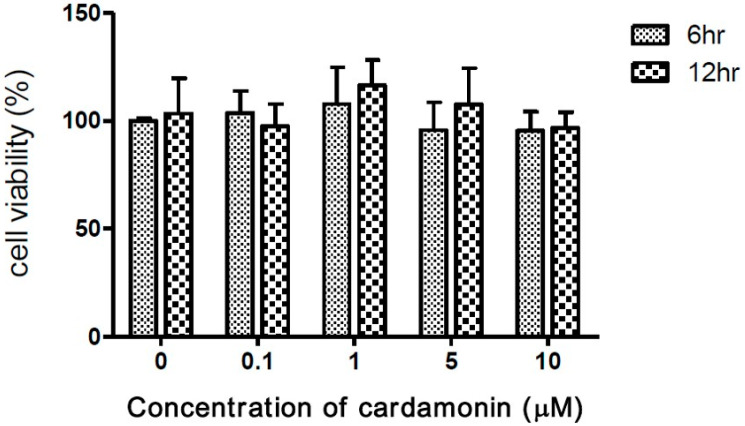
Effects of cardamonin on viability of J774A.1 macrophage cells following incubation with 0–10 μM of cardamonin for 6 or 12 h. Each value represents the mean ± SD of four independent experiments.

Researchers have demonstrated the inhibitory effects of cardamonin on tumorigenesis [25]. To assess the safety of macrophages, experiments were conducted on macrophage cells exposed to cardamonin at concentrations of 0, 0.1, 1, 5, and 10 μM for periods of 6 or 12 h. In 3-(4,5-dimethylthiazol-2-yl)-2,5-diphenyl tetrazolium bromide (MTT) assays, the presence of cardamonin was shown not to significantly affect cell viability at any of the above concentrations (Figure 1).

### 3.2. Cardamonin Inhibited NLRP3 Inflammasome Activation in MSU-Stimulated Macrophages

**Figure 2:**
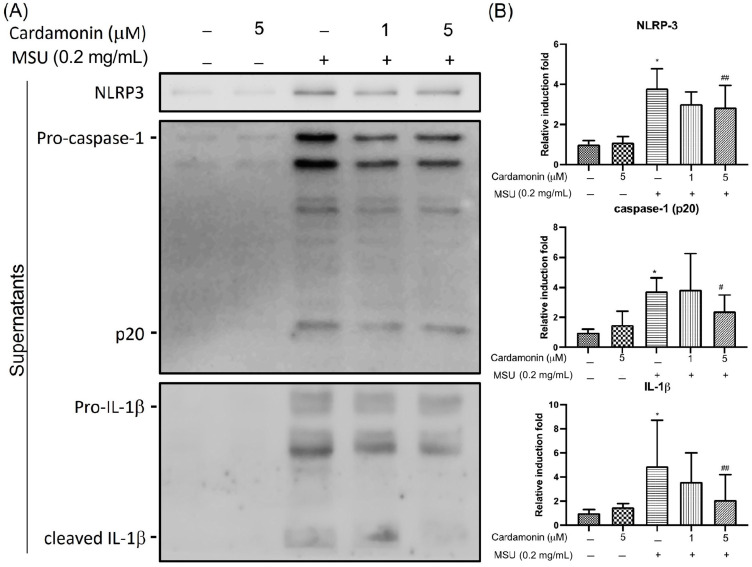
Effects of cardamonin on MSU-stimulated NLRP3 inflammasome activation in J774A.1 macrophage cells treated with MSU for 6 h in the presence or absence of cardamonin: (**A**) Representative gel showing the effect of cardamonin on MSU-stimulated cells. (**B**) Western blot analysis used to detect levels of NLRP3, caspase-1, and IL-1β in supernatants. (–) not treatment; (+) treatment. Each value represents the mean ± SD. Statistical differences among the MSU groups were compared against the control values, whereas cardamonin groups were compared against the MSU group using the ANOVA test (*n* = 5; * *p* < 0.05 vs. control; ^#^ *p* < 0.05 vs. MSU; ^##^ *p* < 0.01 vs. MSU).

Previous research has shown that MSU crystals can induce rapid production of the pro-IL-1β protein. To determine whether cardamonin affects inflammasome-specific cytokine expression, we used Western blot analysis to measure the secretion of NLRP3, IL-1β, and caspase-1 in cell culture supernatants of MSU-stimulated J774A.1 macrophages. NLRP3 expression levels in culture supernatant significantly increased after MSU stimulation, far exceeding levels in the control group. We also compared IL-1β and caspase-1 levels in culture media after stimulating J774A.1 macrophage cells with MSU crystals at a concentration of 0.2 mg/mL for 6 h (Figure 2A). Our results revealed a significant increase in IL-1β and caspase-1 expression. As expected, the activation of NLRP3 via MSU stimulation resulted in the release of mature IL-1β and caspase-1 as well as pro-IL-1β and pro-caspase-1 (p45) into the macrophage supernatant (Figure 2B). We also examined the activation of NLRP3 following exposure to cardamonin at two different concentrations (1 and 5 μM). These results revealed that co-treatment with cardamonin and MSU significantly decreased the secretion of IL-1β and caspase-1 compared with MSU stimulation only, in a dose-dependent manner. In addition, co-treatment with cardamonin (5 μM group) was shown to reverse the MSU-stimulated release of NLRP3.

**Figure 3:**
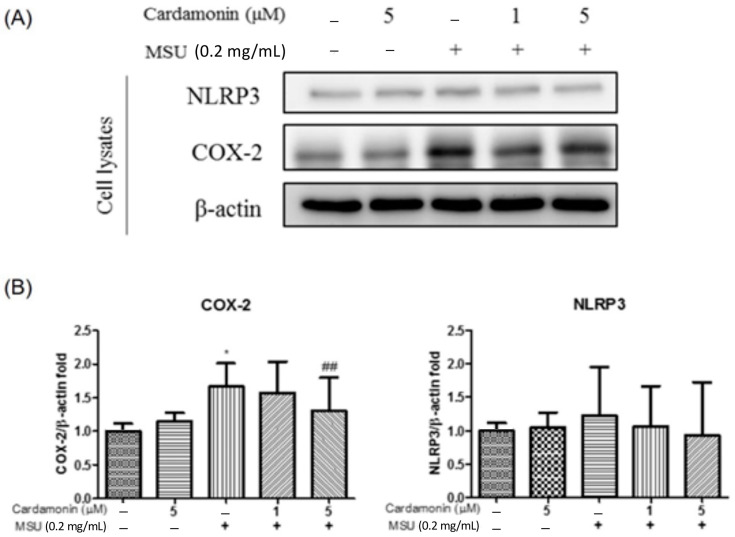
Effects of cardamonin on MSU-stimulated NLRP3 inflammasome activation in J774A.1 macrophage cells. (**A**) Levels of COX-2 and NLRP3 expression in cell lysates. (**B**) Western blot analysis results aimed at detecting the levels of NLRP3 and caspase-1 in cell lysates. (–) not treatment; (+) treatment. Each value represents the mean ± SD. Statistical differences among the MSU groups were compared against the control values, whereas the cardamonin groups were compared against the MSU group using the ANOVA test (*n* = 5; * *p* < 0.05 vs. control; ^##^ *p* < 0.01 vs. MSU).

Western blot analysis was also used to examine the lysate of J774A.1 macrophage cells for indications of COX-2 and NLRP3 expression (Figure 3A). COX-2 expression was significantly higher in the MSU-stimulated group than in the control group. Similarly, NLRP3 expression levels were consistently higher in the MSU-stimulated group than in the control group. We also examined the effectiveness of cardamonin in preventing the expression of COX-2 and NLRP3 in cell lysates by treating MSU-stimulated J774A.1 macrophages with cardamonin. Overall, the expression of COX-2 was significantly lower in the 5 μM cardamonin group than in the MSU-stimulated group. No significant differences were observed between the two groups in terms of NLRP3 expression (Figure 3B).

Taken together, these results indicate that most NLRP3 was passively released under MSU stimulation and that cardamonin decreased the activity of caspase-1 and strongly decreased the secretion of IL-1β in J774A.1 macrophages under MSU stimulation.

### 3.3. Cardamonin Suppressed Gouty Inflammation In Vivo

**Figure 4:**
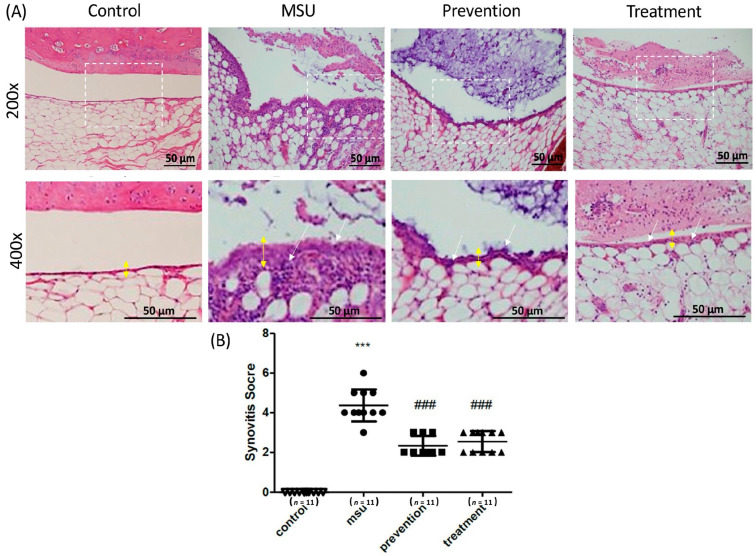
Histopathological analysis of rat knee joints in the four treatment groups, showing the effects of cardamonin on MSU crystals injected into the knee joint cavity: (**A**) Representative images showing H&E histological staining results (200× and 400×). Yellow double arrow: thickness of the synovial layer; white arrow: infiltration cells. Scale bars: 50 μm. (**B**) Quantification of synovitis scores in the four groups including MSU, prevention, treatment, and control groups (*n* = 11 in each group; *** *p* < 0.001 vs. control; ^###^ *p* < 0.001 vs. MSU).

A rat model with intra-articular injections of MSU crystals was used to determine the effects of cardamonin on gouty arthritis in vivo. Rats were divided into four groups: (1) control group (injected with PBS as a control); (2) MSU group; (3) prevention group (intraperitoneal injection of cardamonin every day for a period of five days prior to injection of MSU crystals); and (4) treatment group (intra-articular injection of MSU followed 6 h later by an intraperitoneal injection of cardamonin, occurring at 18 h prior to sacrifice) (Figure 4A). Here, synovitis was used for assessments, as described previously [29]. The histological analysis of the MSU crystal treatment groups showed the neutrophils-predominant inflammatory exudate in the joint space, as well as the infiltration of inflammatory cells in the synovial tissue, accompanied by mild synovial hyperplasia and edematous change. As expected, injection of MSU crystals significantly increased the thickness of the synovial lining and the infiltration of gouty arthritis. Injection with cardamonin reduced the thickness of the synovial lining to normal levels, thereby preventing the infiltration of MSU-stimulated gouty arthritis into the joints at prevention and treatment groups. As for inflammation, cardamonin reduced the expression of white blood cells in joints. Synovitis scores in the prevention and treatment groups were significantly lower than in the MSU group (Figure 4B). Overall, cardamonin had a profound effect in attenuating the inflammation induced by MSU crystals.

## 4. Discussion

Gouty arthritis is the most common form of inflammatory arthritis. According to the 2020 American College of Rheumatology Guidelines for the Management of Gout, the impact of gouty arthritis differs as a function of sex, race, and/or the presence of comorbidities [33]. Chronic gouty arthritis is characterized by intense inflammation, persistent joint abnormalities, and in some cases nephrolithiasis [34]. The initiation of an inflammatory response is caused by the formation and deposition of MSU, leading to the subsequent release of inflammatory mediators [35,36]. Detection of intracellular crystal is essential in diagnosing crystal-induced gouty arthritis because extracellular crystal can be detected as an incidental finding or contamination. At present, the precise mechanism underlying the progression of gout arthritis has yet to be elucidated, and no effective treatment has been devised.

Despite extensive research into the effect of NSAIDs in gouty arthritis, treatment based on chalcone isolates from plants has not previously been assessed. In the current study, the expression level of the NLRP3 inflammasome in MSU-stimulated J774A.1 macrophages was examined following treatment with cardamonin. This research led to three major findings. First, MSU crystals were shown to increase the expression of proteins in the NLRP3 inflammasome in J774A.1 macrophages, including NLRP3 and pro-IL-1β (Figure 2). Second, cardamonin was also found to attenuate gouty inflammation induced by the injection of MSU crystals, in vivo (Figure 4). Third, cardamonin treatment abolished the expression of inflammatory cytokines, for example, COX-2 expression was found to be significantly lower in the 5 μM cardamonin group than in the MSU-stimulated group (Figure 3).

In the current study, MSU crystals induced the expression of inflammatory cytokines and NLRP3 inflammasome activation (including NLRP3 and pro-IL-1β) in macrophages (Figure 2), which is consistent with previous results [8,27]. Previous studies also have demonstrated that the synthesis of pro-IL-1β is the initial priming step in the production of IL-1β [37]. NLRP3 inflammasome activation leads to the conversion of procaspase-1 to active caspase-1, which in turn cleaves pro-IL-1β to form active IL-1β [27]. Evidence obtained in this study suggests that MSU crystal-induced inflammation involves phagocytosis activated by the NLRP3 inflammasome, leading to the secretion of IL-1β from resident macrophages. Our findings also indicate that MSU insult led to NLRP3 inflammasome assembly, caspase-1 activation, and IL-1β secretion (Figure 2). We also observed an increase in the expression of COX-2 in macrophages after insult with MSU crystals (Figure 3). It appears that the therapeutic targeting of the NLRP3 inflammasome may be of benefit to patients with gouty arthritis; however, the effects of cardamonin on the NLRP3 inflammasome in activated macrophages remain poorly understood. NLRP3 inflammasome is a multiprotein complex that is reactive oxygen species (ROS) sensitive. NLRP3 could regulate IL-1β maturation through caspase 1 activation. The result in Figure 2 is assessed from the supernatant. The evidence indicated that MSU could increase the extracellular NLRP3 level to induce extracellular IL-1β maturation. On the other hand, cyclooxygenase-2 (COX-2) is commonly induced by some stimulator, such as MSU. A previous study indicated that COX-2 could regulate NLRP3-derived IL-1β maturation. Here, our results are consistent with the findings from a previous study (Figure 2) [38]. However, COX-2 upregulation did not affect the NLRP3 level in the cell lysate from our result (Figure 3). We could not conclude that COX-2 might directly influence NLRP3 expression or secretion. Furthermore, whether IL-1β might possess the potential to regulate COX-2 expression had been studied [39]. In this study, MSU is the main factor to induce COX-2 expression, not IL-1β. However, COX-2 and IL-1β were involved in many inflammation responses and might have triggered a worse situation under MSU stimulation. Therefore, cardamonin could reduce the level of NLRP3, caspase 1, IL-1β, and COX-2 under MSU stimulation, which might help for gout treatment. Together with other results, our findings provide new insights into the role of cardamonin in the pathophysiology of gouty arthritis.

In previous studies, cardamonin was shown to significantly reduce complete Freund’s adjuvant (CFA)-induced rheumatoid arthritis in rats [40]. Cardamonin has also been shown to inhibit LPS-induced activation of the NLRP3 inflammasome and NF-κB [28,41]. Nonetheless, the effects of cardamonin on MSU-stimulated gouty arthritis via the NLRP3 inflammasome signal pathway have not been extensively investigated.

In the current study, co-treatment of J774A.1 macrophage cells with cardamonin and MSU significantly decreased the secretion of IL-1β and caspase-1 in a dose-dependent manner, compared with MSU stimulation only (Figure 2B). The expression of COX-2 was significantly reduced in macrophages of cell lysate treated with cardamonin compared with MSU stimulation only (Figure 3B). However, we did not observe significant differences in the expression of NLRP3 in the cell lysate of MSU-stimulated macrophages with or without cardamonin treatment. The possible mechanism is the NLRP3 inflammasome that exists in cells, and a small population of cells had apoptosis after the cells were stimulated by different concentrations of MSU. Therefore, the NLRP3 inflammasome in the cell was partially released to the supernatant, causing the expression of NLRP3 inflammasome to be insignificant. Identifying the potential issues underlying NLRP3 expression in cells will require further research. Finally, MSU injection in an orthotopic gouty arthritis model was used to evaluate the in vivo anti-inflammatory activity of cardamonin (Figure 4A). Cardamonin significantly reduced the thickness of the synovial lining and the infiltration of gouty arthritis (Figure 4B). Thus, cardamonin may serve as a preliminary clue for the reduction of inflammation in cases of NLRP3 inflammasome-related disease.

## 5. Conclusions

Along with other results, our findings reveal that cardamonin exerts anti-inflammatory effects by inhibiting activation of the NLRP3 inflammasome in J774A.1 macrophages in vitro. Importantly, cardamonin significantly attenuated IL-1β secretion, caspase-1 activation, and COX-2 production stimulated by MSU crystals. On the other hand, cardamonin had a profound effect in attenuating the inflammation induced by MSU crystals using a gouty arthritis model, in vivo. These findings provide new insights into the molecular mechanisms underlying the effects of cardamonin treatment for gouty arthritis.

## Figures and Tables

**Table 1:** Histopathological assessment of synovitis score.

**Enlargement of Synovial Lining Cell Layer**
0 point Thickness 1–2 cells
1 point Thickness 2–3 cells
2 point Thickness 3–5 cells
3 point Thickness >5 cells
**Inflammatory Infiltrate**
0 point No inflammatory infiltrate
1 point Few mostly perivascular situated lymphocytes or plasma cells
2 point Numerous lymphocytes or plasma cells, sometimes forming follicle-like aggregates
3 point Dense band-like inflammatory infiltrate or numerous large follicle-like aggregates

## Data Availability

Not applicable.
